# McGill University

**Published:** 1895-03

**Authors:** J. M. Parker

**Affiliations:** Secretary


### McGILL UNIVERSITY.

*To the Dean and Faculty of Comparative Medicine and Veterinary Science, McGill University, Montreal.*

GENTLEMEN: The Alumni of the Montreal Veterinary College practising in the State of Massachusetts, U. S. A., held a meeting in Boston on the 2d day of February, 1895.

The names of those present were: Thomas Blackwood, Class '76; James McLaughlin, Class '77; Charles Winslow, Class '79; B. D. Pierce, Class '81; W. P. Mays, Class '85; James B. Paige, Class '88; C. R. Simpson, Class '88; John Roberts, Class '88; W. M. Simpson, Class '89; J. M. Parker, Class '89; Charles Lamb, Class '89: D. L. Bolger, Class '92; George H. Lee, Class '92.

Dr. D. McEachran, Montreal, and Dr. J. W. Gadsden, Philadelphia, were present as guests.

During the meeting it was

*Resolved*, To respectfully urge on the faculty the desirability of adding to their course of instruction a post-graduate course, so as to give the Alumni an opportunity of further prosecuting their studies in the higher branches of medical science under the improved advantages now offered and to be offered by the increased facilities of study at the University.

THOMAS BLACKWOOD, D.V.S.
JAMES B. PAIGE, D.V.S.
JOHN M. PARKER, D.V.S.
Committee.

Many other matters of interest to the profession were discussed, and added to the pleasure of the gathering.

Dr. J. R. McLaughlan, of Newton, was eleeted President, and Dr. J. M. Parker, of Haverhill, Secretary for the ensuing year.

DR. J. M. PARKER,
Secretary.

---

THE annual commencement exercises of the American Veterinary College take place March 25, 1895.

The Chicago Veterinary College, March 21, 1895.

